# Local overfishing may be avoided by examining parameters of a spatio-temporal model

**DOI:** 10.1371/journal.pone.0184427

**Published:** 2017-09-08

**Authors:** Stuart Carson, Nancy Shackell, Joanna Mills Flemming

**Affiliations:** 1 Department of Mathematics and Statistics, Dalhousie University, Halifax, Nova Scotia B3H 3J5, Canada; 2 Bedford Institute of Oceanography, Fisheries and Oceans Canada, P.O. Box 1035, Dartmouth, Nova Scotia B2Y 4T3, Canada; Maurice Lamontagne Institute, CANADA

## Abstract

Spatial erosion of stock structure through local overfishing can lead to stock collapse because fish often prefer certain locations, and fisheries tend to focus on those locations. Fishery managers are challenged to maintain the integrity of the entire stock and require scientific approaches that provide them with sound advice. Here we propose a Bayesian hierarchical spatio-temporal modelling framework for fish abundance data to estimate key parameters that define spatial stock structure: persistence (similarity of spatial structure over time), connectivity (coherence of temporal pattern over space), and spatial variance (variation across the seascape). The consideration of these spatial parameters in the stock assessment process can help identify the erosion of structure and assist in preventing local overfishing. We use Atlantic cod (Gadus morhua) in eastern Canada as a case study an examine the behaviour of these parameters from the height of the fishery through its collapse. We identify clear signals in parameter behaviour under circumstances of destructive stock erosion as well as for recovery of spatial structure even when combined with a non-recovery in abundance. Further, our model reveals the spatial pattern of areas of high and low density persists over the 41 years of available data and identifies the remnant patches. Models of this sort are crucial to recovery plans if we are to identify and protect remaining sources of recolonization for Atlantic cod. Our method is immediately applicable to other exploited species.

## Introduction

Fish are not randomly distributed across a seascape. They occur at higher concentrations in habitat where resources can support higher densities. The geographic distribution of fish may either expand in proportion to an increase in abundance, or it may exhibit a density-dependent habitat selection response, in which fish preferentially occupy a preferred area until it reaches maximum density, at which point they disperse into more marginal areas [1–3]. Under either conception of the range expansion process, core areas are occupied at low population size [1]. The series of ‘core’ or high-density areas across the seascape can be considered a meta-population, a series of sub-populations that are connected to a greater or lesser degree, and where geographically closer, sub-populations are relatively more connected [4].

Fishing boats naturally tend to focus on high density core areas, in order to minimize effort and maximize catch. For fish species that select or occupy habitat based on density, any core area that gets depleted by fishing will fill up with fish from surrounding areas. Fishing can continue, until there are insufficiently many fish to move in and maintain density, the area then becomes locally depleted. This process has been referred to as local overfishing [5] and if it happens often, the species experiences spatial erosion across the seascape. The consequences of local overfishing can lead to stock collapse [5–8]. Sufficient evidence of spatial erosion and non-recovery has accrued [9–12] that fishery managers are becoming interested in how to maintain the integrity of a stock’s spatial pattern. Here we propose a Bayesian hierarchical spatio-temporal model that involves 3 key parameters to describe spatial structure: persistence (similarity of spatial pattern over time), connectivity (degree of coherent structure present) and spatial variance (variation across the seascape). Our goal is to show that these parameters can be well estimated to provide a useful picture of stock structure on both long-term and annual scales. The long-term model parameter estimates (persistence, connectivity, spatial variance) can be interpreted as the climatological, or average spatial structure. We examine the behaviour of these parameters from the height of the fishery through the collapse. Further, our model framework can be used on an annual scale to monitor and potentially maintain a stock’s spatial structure. We use Atlantic cod (Gadus morhua) as a case study, a well known fish species with a long history as a commercially valuable and widely consumed food fish.

The fishery for Atlantic cod in eastern Canadian waters has a history dating back several centuries but has perhaps been most widely recognized in recent years for the closure of the fishery due to the collapse of the exploited stocks that occurred in the early 1990s [13–17]. One feature of these stock collapses was the very late realization that the stocks were in peril; catches, and inferred stock levels, remained high right up until the seemingly sudden and precipitous collapse [18]. Stock assessments may have missed the signs of the impending collapse because the distribution of cod throughout the northwest Atlantic can be density dependent [19–22] making cod susceptible to being locally overfished. Specifically, it is suggested that there exists a region of prime habitat or ‘core range’ and that this prime range is used preferentially and that the stock’s total range extends out from the core into less preferred areas under population pressure in what is termed an Occupancy-Abundance relationship; range is positively related to abundance [1, 2], and the species is said to display Density Dependent Habitat Selection (DDHS) [23, 24]. The actual density of the species of interest in the prime habitat may remain relatively constant even as the total abundance reduces if there is in-migration from the less preferred range. In the case of the Northern cod, the fishing industry’s standardized measure, catch per unit effort (CPUE) remained high, but in reality these cod were becoming spatially concentrated [13, 18, 19] as abundance decreased. The effort expended to obtain a profitable trawl remained fairly constant but the area of ocean where these profitable trawls were being found was becoming smaller and smaller [25]. Our study contributes to the growing effort to develop spatial indices that will help to maintain stock spatial integrity [26].

## Data

**Fig 1:**
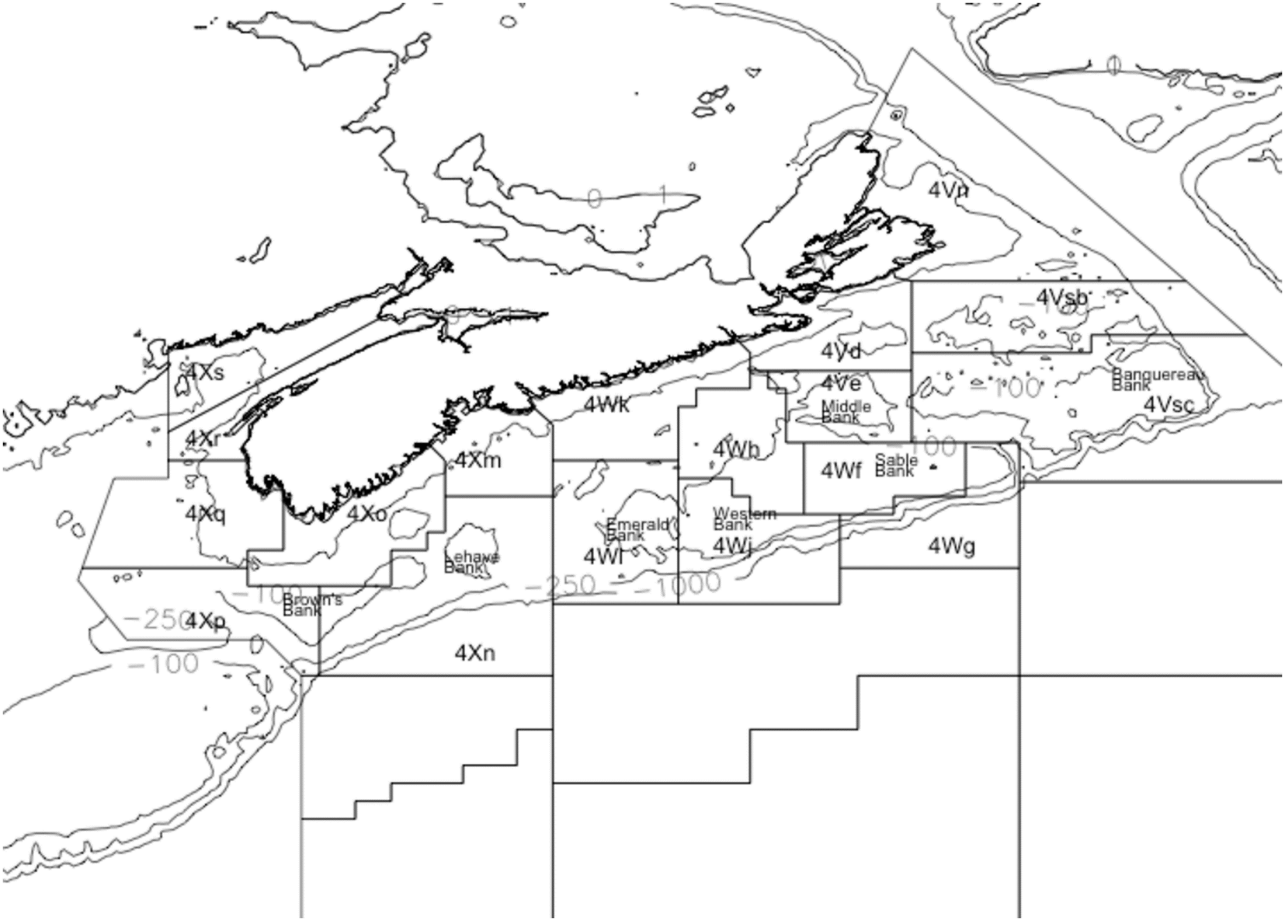
NAFO areas and named features. Designations of the NAFO zones and sub-zones on and around the Scotian shelf; 4X, 4W and 4V. The area within the contour lines marks the approximates the Scotian Shelf.

The Department of Fisheries and Oceans Canada (DFO hereafter) Maritimes Region has conducted an annual summer ground fish research trawl study each year since 1970. Originally designed to measure distribution and abundance of commercial species these data also incorporate information on non-commercial species. Focussed upon the Scotian Shelf the DFO survey utilizes a stratified sampling plan using the three relevant North American Fisheries Organization (NAFO) zones, 4V, 4W, and 4X demarcating the Scotian Shelf. Fig 1 presents the general geographical location and shows the boundaries of these NAFO areas and their associated sub-divisions, referred to as sub-zones. Each of the three NAFO zones has sampling effort (the number of sample trawls) proportional to their area. The catch is sorted by species, weighed and measured for individual weight, maturity status and age. The data have been summarized in various reports [27–29], stored, and are publicly available in the Ocean Biogeographic Information System (OBIS) [30]. OBIS is the DFO—Maritimes Region database for ground fish research trawl surveys and includes information on some 263 distinct species found on the Scotian shelf. It includes descriptive data for each cruise or mission resulting in about 200 fishing sets per year. For each set there exists trawl information: date, latitude, longitude, distance towed(km), as well as physical/water characteristics at the location and depth of the trawl; temperature(C), salinity(ppm), nitrate(ppm), phosphate(ppm) and silicate(ppm). For all species captured: genus, species, common name, total weight(kg) and count, (and total weight and count standardized by distance towed) are recorded.

Here we consider a single species, Atlantic cod, but stress that our methodology can be routinely applied to any species. We take as our response variable Atlantic cod abundance with the objective of demonstrating how a Bayesian hierarchical spatio-temporal model brings forward, in a novel and yet easy to visualize way, what, and when, the cod population did with respect to distribution and abundance during the critical years of 1986 through 2003. These are the years for which we have both the OBIS trawl data and the best available fishing data for Atlantic cod which are annual landings. These landings are not spatially indexed, that is, the locations where the cod were harvested are not known, only the NAFO sub-zone (in some cases only zone) was recorded. We therefore calculate landings by sub-zone by year in an effort to assess the impact of fishing directly. Specifically we utilize a single number summary for cod landings by year and sub-zone, where for sub-zone(i), the landings for that year are calculated (*Landings*_*i*,*t*_) as well as the total landings for the year (*Landings*_*t*_). These are combined with our measure of abundance, the OBIS trawl data, which too is summed by sub-zone (*OBIS*_*i*,*t*_) and by year (*OBIS*_*t*_). The *relative exploitation* rate (RE) in that sub-zone is then calculated as,

$$\begin{array}{c} RE\left(i,t\right)=\frac{\frac{Landings_{i,t}}{Landings_{t}}}{\frac{OBIS_{i,t}}{OBIS_{t}}}. \end{array}$$ (1)

These data allow us to explore the relationship between the response and the available cod landings data since it is commonly held that the root cause of the cod stock collapse was overexploitation [13, 15, 16, 31]. We have quite a long time series of data, 1970 to 2014, and some knowledge of what the nature of the fishing pressure was on Atlantic cod. Broadly this timespan may be separated into four distinct periods, based on the nature of the fishing pressure:

1. In the first period, 1970-1977, the main fishing effort was by the foreign fleets. It is widely held that overfishing by these foreign flagged vessels was responsible for overfishing the Atlantic cod precipitating the ‘first collapse’ in 1975.
2. In the second period, 1978-1985 the Cod experience a rebound in abundance as, after the imposition of a Canadian 200 nautical mile Exclusive Economic Zone(EEZ), the Atlantic cod stock was under lessened fishing pressure since the ‘foreign fleet’ was no longer operating in the new EEZ.
3. In the third period, 1986-1992, the Canadian domestic fleet ramped up to fill the void left by the departing foreign vessels and the fishing pressure upon the Atlantic cod stock re-intensifies, leading to another, this time even more pronounced, ‘second collapse’, followed by the eventual imposition of the moratorium.
4. In the fourth period, 1993-Present, the Atlantic cod stock remains at very low historical levels, and, despite the cessation of fishing, has ‘failed to recover’ its former abundance.

There are several other covariates worthy of consideration. In addition to the covariates measured at the time of the trawl (e.g. temperature) we also consider an oceanographic covariate (Bathymetry). Bathymetry for the area of the Scotian Shelf has been obtained from the U.S. National Oceanographic Atmospheric Administration (NOAA) [32].

## Methods

Spatial models depend on Tobler’s Law of geography, which states that all locations are related but neighbouring locations are more related than distant locations, and estimate a statistical correlation in the residuals, after accounting for the effect of covariates [33]. We, and others, find Gaussian random fields (GRFs) [34] to be the simplest full implementation of spatial modelling. GRFs can be efficiently used to simulate spatial dependencies in order to estimate spatial correlations in a statistical model [35], i.e. the covariance matrix, Σ, and express these with a simple and interpretable set of parameters *ρ*, *σ*, which we interpret as connectivity and spatial variance respectively. A third parameter, *a*, here referred to as persistence, arises if a temporal structure is desired. For small sample sizes Σ can be calculated to estimate these parameters directly. However, this requires inverting Σ, which becomes computationally infeasible for a large number of points. INLA, (for Integrated Nested Laplace Approximation) [36, 37], approximates the inverse-covariance matrix, (Σ^−1^), of the GRF using sparse matrices calculated using the stochastic partial differential equation approach [38, 39]. This approximation is extremely fast, and is easily implemented using R-INLA [36] in the R statistical platform [40]. Given the ease, efficiency, and generality of the R-INLA package, we propose a Bayesian Hierarchical Spatio-Temporal model framework for the Atlantic Cod abundance data. This approach has been used in animal tracking [39, 41] and more recently in the marine context [42, 43], modelling habitat [44], nurseries [45], and bycatch [34, 46, 47].

### Spatio-temporal model structure

The response variable *y*(*s*, *t*) is the total number of cod captured in a single trawl (1 to a maximum observed count of 12189) at location *s* at time point *t*, *t* ∈ (1986, …, 2003). Since these data are counts, we consider suitable candidate distributions including the Poisson and negative binomial distributions (each with their respective canonical link functions [48]).

The mean of our response, *E*[*Y*(*s*, *t*)] = *μ*(*s*, *t*), is mapped by a link function to a linear predictor *η*(*s*, *t*) as in the Generalized Additive Model framework [49]. That is,

$$\begin{array}{c} \eta \left(s,t\right)=\xi \left(s,t\right)+\sum_{j=1}^{n_{f}}f_{j}\left\{c_{j}\left(s,t\right)\right\}, \end{array}$$ (2)

where the linear predictor is the sum of parts; a spatio-temporal random effect *ξ*(*s*, *t*), and smoothed functions of covariates *f*_*j*_{*c*_*j*_(*s*, *t*)}, where *n*_*f*_ is the number of covariates. The *f*_*j*_{*c*_*j*_(*s*, *t*)} are smoothed functions of covariates rather than linear ones, where *c*_*j*_(*s*, *t*) is the value of the *j*th covariate at location *s* and time *t*. Using such functions allows the effect of the covariate to vary across its values. Several of the potential covariates are highly co-linear, such that it would be inappropriate to include all of them in our model framework simultaneously. For covariates that have pairwise correlations ≥ 0.9 (e.g., nitrate and silicate), we consider only models that contain one or the other. The spatio-temporal random effect *ξ*(*s*, *t*) may be thought of as representing the cumulative effect of latent factors impacting the response and so can be interpreted as a latent variable [41] where its characteristics compose the spatial and temporal covariance structure of the model, here that of the Atlantic cod distribution on the Scotian shelf.

#### GMRFs and the SPDE approach

GRFs are usually defined by a mean and a covariance function *Cov*[(*s*, *t*), (*s*′, *t*′)] defined for each (*s*, *t*), (*s*′, *t*′) in *R*^2^ × *R*, that is, defined between locations(*s*) and times(*t*). Modelling Gaussian fields directly is often difficult, especially for large data sets and there is some literature on this problem [50–52]. The Stochastic Partial Differential Equation (SPDE) approach, in which a spatio-temporal random effect *ξ*(*s*, *t*) is treated as a GRF and represented with a Gauss Markov Random Field (GMRF), is one attempt to surmount this difficulty with some computational simplifications. Under the SPDE approach, the continuously indexed GRF is represented as a *discretely* indexed random process, a GMRF. The computational advantages are realized by this representation since the continuous integrals of the GRF are replaced by the discrete sums of the GMRF. A thorough explanation, proofs and theoretical details may be found in [38].

Let us, for explanatory purposes, consider our penultimate model. This model will incorporate a first order auto-correlated spatio-temporal effect between years with coefficient *a*. This means that the random field incorporates a temporal persistence parameter, (|*a*| < 1), combined with a spatial covariance function. That is,

$$\begin{array}{c} \xi (s,t)=a\xi (s,t-1)+\omega (s,t) \end{array}$$ (3)

and,

$$\begin{array}{c} Cov\left[\omega \left(s,t\right),\omega \left(s^{\prime },t^{\prime }\right)\right]=\{\begin{array}{cc} 0 & \text{if}\;t\neq t^{\prime }\\ \sigma_{\omega }^{2}C\left(h;\nu ,\kappa \right) & \text{if}\;t=t^{\prime }, \end{array} \end{array}$$ (4)

where *ω*(*s*, *t*) has a zero mean gaussian distribution, is temporally independent, and has a spatial covariance function for *s* ≠ *s*′ where

$$\begin{array}{c} C\left(h;\nu ,\kappa \right)=\frac{1}{\Gamma \left(\nu \right)2^{\nu -1}}{\left(\kappa h\right)}^{\nu }K_{\nu }\left(\kappa h\right). \end{array}$$ (5)

The parameters of this Matérn covariance function, *C*(*h*;*ν*, *κ*), are *ν* and *κ*, *ν* > 0, *κ* > 0. (*K*_*ν*_ is the modified Bessel function of the second kind). The parameter *ν* determines smoothness and *κ* determines spatial scale. and the covariance function is a function of the distance separating the locations *h* = ‖*s* − *s*′‖. In practice, the parameter *ν* is usually fixed, (we take *ν* = 1), and $\rho =\frac{\sqrt{8\nu }}{\kappa }$ is reported empirically with *ρ* being the distance at which the spatial correlation is reduced to approximately 0.1 [53] [54].

**Fig 2:**
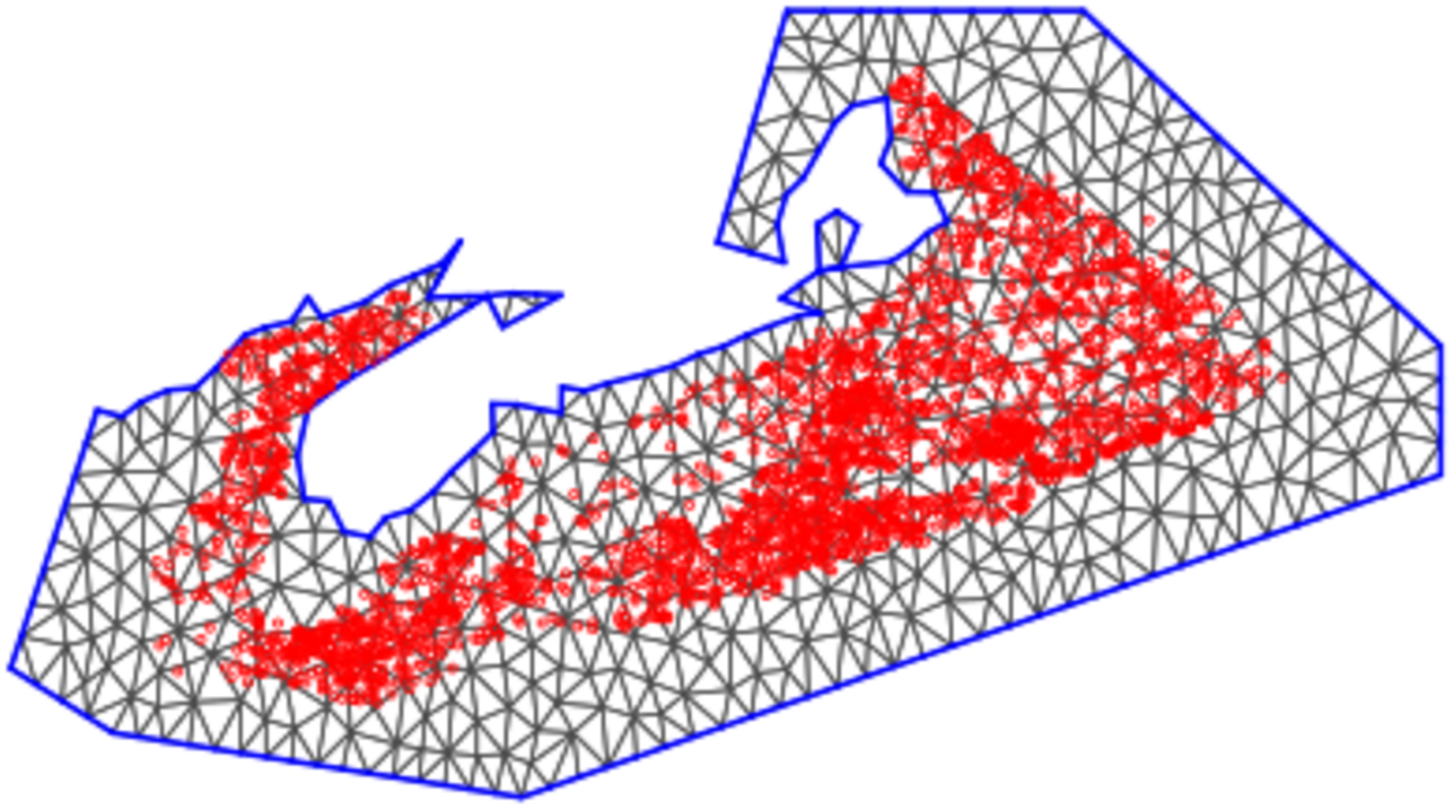
Triangulation. The triangulation utilized for the SPDE approach with n = 548 vertices. Red dots are the data locations.

We have a continuous GF that we want to represent as a GMRF. The GMRF is a spatial process that models spatial dependence on a grid or lattice or graph [39]. If we denote this continuously indexed GF with Matérn covariance function, defined by parameters *κ* and *ν*, as *X*(*s*), the aim is to find a GMRF that best represents *X*(*s*). As an alternative to using a regular grid, the SPDE approach utilizes a triangulation of the domain [38]. The distinction is an important one; the use of a triangulation contributes to the computational advantage of this approach since, unlike a grid, it allows for cells of different sizes, reducing the number of empty cells where data is sparse while retaining fine resolution where data is dense. The domain is subdivided into non-intersecting triangles with vertices at the data locations. Additional vertices are then added sufficient to get a useful triangulation. Some care is required in the process of creating and defining a mesh, since it is desirable to have a mesh with triangles of somewhat similar size and shape, while avoiding any excessively acute vertices, [38]. The R-INLA package offer some tools to assist the practitioner in achieving a suitable mesh. The ‘max.edge’ tool allows the user to specify the maximum side length for a triangle (and thus limit the maximum triangle size and hence resolution of the mesh), while the ‘cutoff’ tool allows the user to treat data points within a specified distance to be treated as one point, thus preventing overly small triangles and so controlling the minimum resolution of the mesh. Our triangulation is shown in Fig 2.

Very simply, the SPDE will represent *X*(*s*) at each vertex and interpolate values in between. More completely, the basis function representation of the original field *X*(*s*) is;

$$\begin{array}{c} X\left(s\right)=\sum_{l=1}^{n}\psi_{l}\left(s\right)\epsilon_{l}, \end{array}$$ (6)

where *n* is the number of vertices in the triangulation, *ψ*_*l*_(*s*) are the basis functions and *ϵ*_*l*_ are gaussian weights. The basis function *ψ*_*l*_(*s*) is equal to 1 at vertex *l* and 0 at all other vertices. The value of the field at any vertex is given by *ϵ*_*l*_ and values for the interior of the triangles are determined by linear interpolation. Once written this way [38] show that there is a mapping of the covariance function *C*(*h*;*ν*, *κ*) of the Gaussian field to the covariance matrix of the GMRF, (through its inverse, the precision matrix, *Q* = Σ^−1^), such that the spatio-temporal model can be rewritten in terms of a GMRF.

### Model assessment

In a Bayesian approach, the parameters that comprise our model are treated as random variables and prior information about the parameters is incorporated in a prior distribution. Recently, INLA has expanded the prior options it offers the analyst. INLA has incorporated an methodology for prior selection using ‘penalized complexity priors’ (pc.prior) [55]. This construction, which seeks to provide weakly informative default priors that “are useful, understandable, conservative, and better than doing nothing at all“ [55]. In the kind of model we have here, the random field is a spatial random effect; if there is no spatial random effect it is equivalent to having *ρ* = ∞ and *σ* = 0, that is, the effect is a cnstant 0 everywhere. Having a finite *ρ* and *σ* > 0 makes the model more complex, hence the rationale. The pc.prior format allows the user to control the priors by considering the problem. The user is required to supply a value for *ρ*_0_ and a probability that *ρ* < *ρ*_0_. By considering a reasonable lower value for the spatial effect *ρ*_0_ is chosen. The probability chosen supplies the weight of the penalty on the more complex model. For *σ*, one considers a reasonable upper value for the spatial variance, the penalty shrinks the model toward *σ* = 0, since that is the simpler case. With no a priori expectation, we chose a values of *ρ*_0_ = 0.5 with *P*(*ρ* < 0.5) = .5. By a similar process we chose *P*(*σ* > 0.75) = .5. All the models we subsequently report use these priors.

The various candidate models are compared using the Deviance Information Criterion (DIC), the Logarithm of the Pseudo Marginal Likelihood (LPML) and/or the Root Mean Squared Estimation Error (RMSEE).

#### Deviance Information Criterion: DIC

The DIC [56] is the most common diagnostic function found in discussion of Bayesian models. It works by summing a quantity, the expected deviance *E*[*D*(*θ*, *x*)], with another, the number of effective parameters *p*_*D*_. Models with *lower* sums, (lower DIC), are considered superior. The DIC is calculated by INLA by default and is found in the model output from an INLA model. To simplify interpretation, DIC measures the goodness of model fit while simultaneously penalizing complex models.

#### Logarithm of the Pseudo Marginal Likelihood: LPML

Another Bayesian diagnostic model criterion is the Conditional Predictive Ordinate (CPO) [57], a *W-fold* leave one out cross validation. This is calculated by taking *W* equal sized samples, (typically 5 or 10 percent of observations, here 5) from the data, *x*_1_, …, *x*_*w*_, calculating an estimate for each location (*s*, *t*), and for each location averaging over the samples to find $\hat{CPO}$ as:

$$\begin{array}{c} {\hat{CPO}}_{\left(s,t\right)}={\left(\frac{1}{W}\sum_{w=1}^{W}\frac{1}{\pi (y_{\left(s,t\right)}|x_{w},\theta_{w})}\right)}^{-1}, \end{array}$$ (7)

Now, *CPO*_(*s*, *t*)_ is a goodness of fit measure for each observation—it can be summarized for the entire data set as a single value, LPML, with *y*_−(*s*, *t*)_ being *y* without the (*s*, *t*) st element.

$$\begin{array}{c} LPML=\sum^{n_{obs}}log\left[\pi \left(y_{\left(s,t\right)}|y_{-(s,t)}\right)\right]\approx \sum^{n_{obs}}log\left[{\hat{CPO}}_{\left(s,t\right)}\right]. \end{array}$$ (8)

In this way the LPML acts as a comparator of the predictive quality of the models. The *larger* the CPO, the better the model. INLA ordinarily calculates the CPO as part of the default output.

#### Root Mean Squared Estimation Error: RMSEE

The closeness of the estimation can be checked via the Root Mean Squared Estimation Error (RMSEE). The RMSEE is not calculated by R-INLA, but is readily calculable from the observations (*y*_(*s*,*t*)_) and the linear predictor from Eq 1.

$$\begin{array}{c} RMSEE=\sqrt{\frac{1}{n_{obs}}\sum d_{\left(s,t\right)}^{2}};\;d_{\left(s,t\right)}=y_{\left(s,t\right)}-E\left[Y_{\left(s,t\right)}|x,\theta \right] \end{array}$$ (9)

Clearly, *smaller* is better.

## Results

Our model framework involves a spatio-temporal covariance structure and a set of covariates that best describe the response (the *ξ*(*s*, *t*) and *c*_*j*_ of Eq 1 respectively). We consider models that include no spatial or temporal effect at all; this amounts to simply modelling the response either as a mean (without covariates) or as a function of the covariates (only). We also consider models with a single spatial effect constant through time, as well as those with temporally varying effects. Temporally varying models considered are those with spatial effects replicated each year, that is, a single spatial effect for each year (without temporal correlation), and models with spatial effects correlated between years via an AR(1) relationship. We select the best spatio-temporal structure the same way as we select the best distribution for *y*(*s*, *t*) and the same way as we choose our eventual covariates. That is, we run models using the various alternative constructions and compare them using the model assessment criteria discussed in the previous section. For all spatio-temporal model formulations considered, the DIC for the negative binomial response distribution was always lower than that of the Poisson (LPML is higher, RMSEE is lower). Hence the negative binomial distribution is to be preferred and for brevity we display results in Table 1 for only for the negative binomial response and the three best performing physical covariates. Amongst these models the inclusion of the AR(1) temporal structure results in the lowest observed DIC (highest LPML, lowest RMSEE). On this basis we choose the AR(1) spatio-temporal structure. This leads to the following model formulation:

$$\begin{array}{c} \eta (s,t)=\xi (s,t)+f(RE(i,t))+f(Temperature(s,t)), \end{array}$$ (10)

where *η*(*s*, *t*) is modelled as in Eq 3, and with smoothed functions of Relative Exploitation and the Temperature at the trawl. This model performed best according to both the DIC and the RMSEE criteria. There was a slight improvement in LPML when including a Bathymetry covariate but this resulted in poorer DIC and RMSEE measures and including Bathymetry along with RE and Temperature did not improve estimation.

**Table 1:** DIC values. DIC values for the various candidate models with an AR(1) spatio-temporal covariance structure.

Covariate(s)	DIC	LPML	RMSEE
*ξ*(s,t) Alone	22342.11	-18244.10	245.58
*ξ*(s,t)+f(Temperature)	22277.74	-18192.46	246.04
*ξ*(s,t)+f(Bathymetry)	22236.59	-18203.59	246.48
*ξ*(s,t)+f(RE)	22166.12	-18173.36	246.39
*ξ*(s,t)+f(Temperature)+f(Bathymetry)	22206.37	**-16145.51**	244.52
*ξ*(s,t)+f(RE)+f(Temperature)	**22131.31**	-16245.66	**240.68**
*ξ*(s,t)+f(Bathymetry)+f(RE)	22138.84	-17520.52	245.77
*ξ*(s,t)+f(RE)+f(Temperature)+f(Bathymetry)	22155.41	-176742.23	251.08

**Fig 3:**
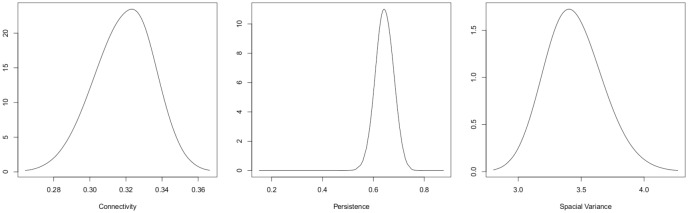
Results. Posterior distributions for the model parameters.

The parameters of the model specify the spatio-temporal random effect. Considering each of these estimates one at a time, *ρ* is the spatial connectivity parameter, the range at which the spatial correlation is reduced to approximately 0.13. That is, the value $\hat{\rho }=0.321$ is the estimated distance (in degrees—so approximately 34 km) at which this occurs. The estimated spatial variance of the GMRF is $\hat{\sigma }=3.39$. The coefficient for the AR(1) process (the persistence parameter, *a*) in Eq 2 is estimated at 0.627. For the negative binomial distribution assumed for the responses, $\hat{\phi }=log\left(n\right)=0.986$ (*n* is the size parameter, $\sigma_{nbin}^{2}=\mu \left(s,t\right)\left(1+\frac{\mu (s,t)}{n}\right)$). Plots of the posterior distributions of the parameters are provided in Fig 3.

**Fig 4:**
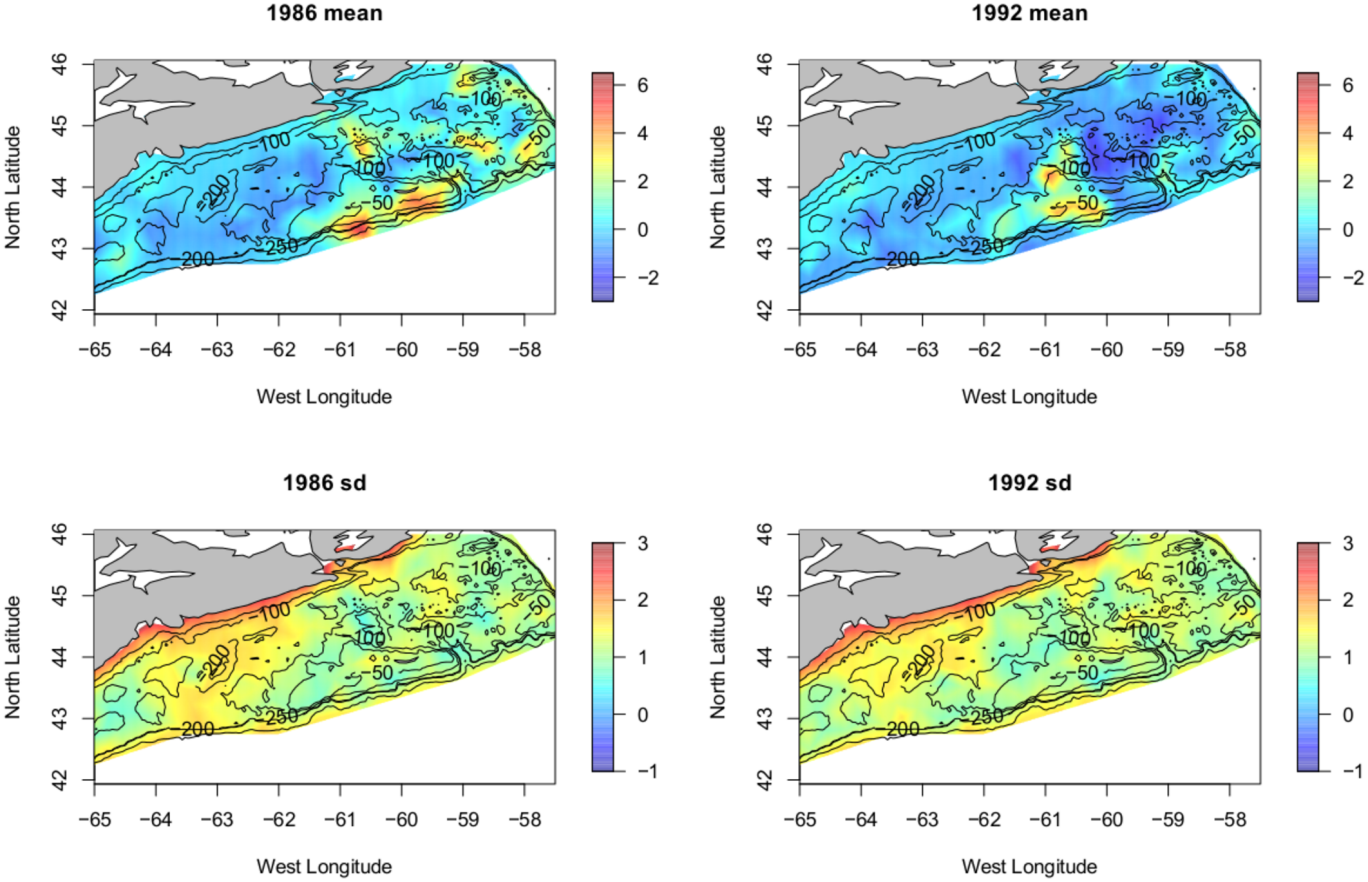
Results. Plot of the posterior mean for the years 1986 and 1992 (pre and post collapse). The lower panels show the corresponding plots of uncertainty (the response SD). The scale is log(predicted count).

**Fig 5:**
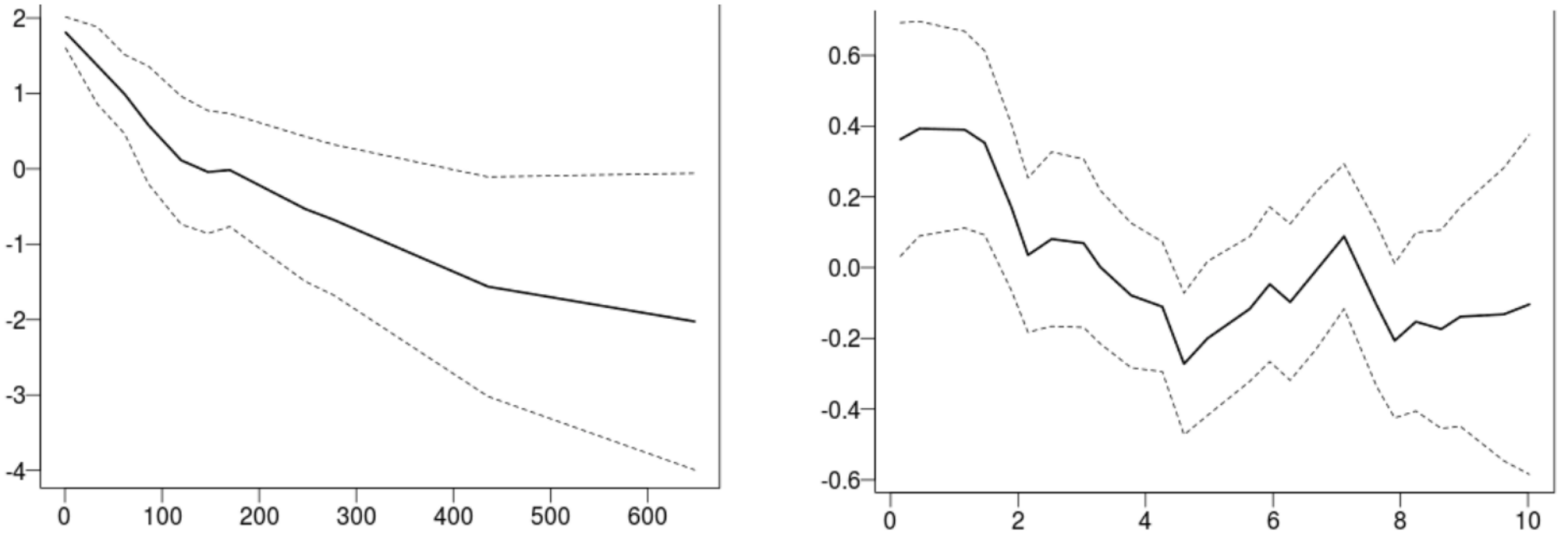
Relative exploitation and temperature effects. The covariate plots show the value of the covariate on the X axis, and the impact of the covariate on the response on the Y axis and their 95% credible intervals (the dashed lines). Viewed as functions of the covariates, lower Relative exploitation and colder temperatures produce higher predicted abundance.

By assembling the above components we can nicely display the results. Combining the effects of the fixed covariates with the model output for the random field gives us the mean of the model for each year. A plot of this mean for 1986 (pre-collapse), is shown in the upper left panel of Fig 4 and consists of the sum of the linear predictors in the model and the elements of the random field. A similarly constructed plot for the year 1992 (post-collapse) is shown in the upper right panel of Fig 4. The estimated functions for the effects of the two covariates are shown in Fig 5.

Relative Exploitation and Temperature are both seen to have some significant effect on cod abundance over portions of their ranges. The dashed lines in Fig 5 indicate the 95% credible intervals for the estimated effects. The covariates have significant effects over those portions of their ranges where this interval does not include zero. When the water is cold (< 2°C) the effect is positive and trends toward negative as water temperatures rise to 10°C. Low relative exploitation levels, (< 100), positively impact abundance. While not significantly non-zero, the trend in these two covariates are in the expected direction, and, these results are entirely consistent with expectations and what is previously known about cod [58, 59].

We also fit our model to each year of data individually (and consequently without the persistence parameter) in order to obtain annual estimates of both the spatial connectivity parameter *ρ* and the abundance parameter *σ* so as to look for patterns potentially related to levels of exploitation. A direction of future research is to consider a single model that incorporates autoregressive relationships (for example) between these parameters, but this generality is not presently available using INLA.

## Discussion

Local over-fishing (serially fishing out concentrations that do not replenish) has been inferred for Northern cod [13, 18, 19]. Our spatio-temporal model approach makes it evident that cod became spatially concentrated as abundance decreased, until they became so depleted that abundance even in the core areas collapsed.

**Fig 6:**
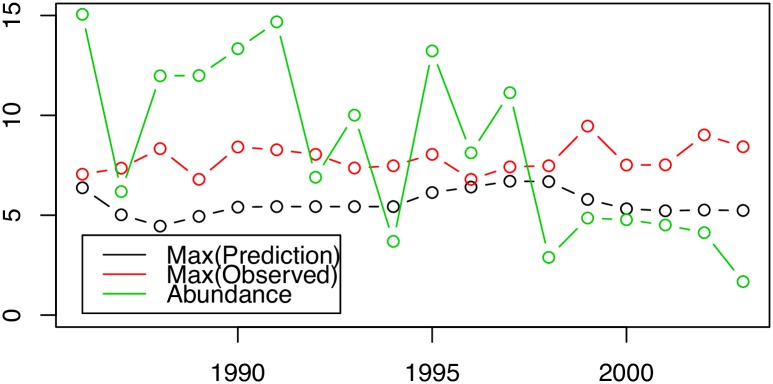
Hyperstability. The maximum *observed* count (LogY), the maximum *predicted* count (eta) are nearly constant, even though the stock is collapsing.

**Fig 7:**
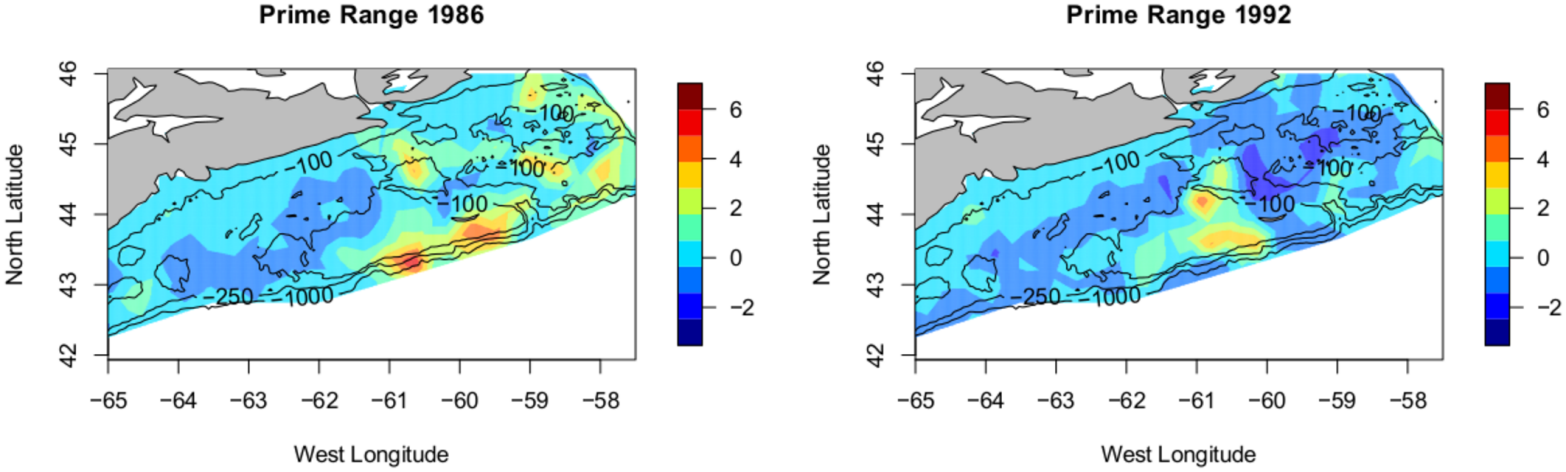
Core range. Plot of the posterior mean for the years 1986 and 1992, showing only those areas where the predicted mean is > 75th percentile. Viewed as ‘core range’. The species range has contracted with the reduced abundance but maximum density in the aggregations has not changed, scale is log(abundance).

We begin by considering out results, pre-collapse vs. post-collapse, as in Fig 4; estimated abundance was very high in 1986 but by 1992 had collapsed precipitously. Looking at the posterior mean of the model for these two years we see that the largest forecast values in 1986 and 1992 (the red areas) are located in (approximately) the same location, (around 60-61W, 43N) and, moreover, have (again approximately) the same predicted value despite the precipitous decline in overall abundance, see Fig 6. Indeed the maxima of the observations and the maxima of the predicted values are similar in value and the value does **not** decline along with the overall decline in abundance seen over this period. What does appear to change is the spatial extent of the moderate values. Away from the red there is a general decline in the predicted values; seen as the areas of pale red/yellow in 1986 appearing as blue in 1992. The decline is seen as a reduction of the spatial extent of high and moderate values, not as a decline in the maximum values. This is entirely consistent with the hypothesized hyper-stability [18] of abundance in the preferred, or *core range* [20]. In order to more fully illustrate what is happening we present two more plots in Fig 7. In this figure only the areas of highest predicted abundance are shown. To emphasize our point, i.e. to highlight those areas where abundance is ‘high’, we chose an (arbitrary) value equal to the 75th percentile of estimated cod abundance values and then plot the locations where cod abundance was predicted to equal or exceed this value. We note that a high AR(1) term tells us that the spatial distribution of biomass stays the same year after year, and we have confidence that high density areas persist and are important. Areas that are always occupied, during periods of high and low abundance are interpreted as ‘core’ areas, but in a collapsed stock, even core areas will disappear [20]. We see that between the left panel and the right panel the area where abundant cod are predicted to be decreases markedly, disappearing completely from previously abundant Banquereau bank, even though peak abundance remains constant. This is interesting as it certainly appears that the cod are contracting towards the areas of highest density as the overall abundance diminishes, another result consistent with previous cod studies, e.g. [19].

In summary our methodology suggests a spatio-temporal model for mean abundance that is entirely consistent with the occupancy-abundance hypothesis. In fact, Figs 4 and 7 illustrate the phenomenon of stable abundance in key areas of the range combined with a contraction of spatial distribution under the circumstances of an overall decline (in this case large) of the population as a whole.

Interpretation of the model parameters themselves is also interesting and indeed entirely consistent with theory. The relatively large value of the persistence parameter in the AR(1) construction, (*a*), suggests a strong connection between the observed cod abundance from year to year; in other words the cod are to be found, or not found, in the same places year after year. Thus the areas of consistently high abundance may be thought to be important to the stock, congruent to the idea of core range posited earlier. The range or scale parameter, *ρ*, is interpretable since it is the distance at which covariance is considered to become insignificant (<0.13). The value $\hat{\rho }=0.321$ is the distance (in degrees) giving us an idea of the physical scale of the cod core range or ranges, 34 km. One way of looking at the meaning of *ρ* = 0.321 is to consider the implication of independence at distances greater than *ρ*. If we have 2 or more areas of consistently high abundance separated by some distance greater than *ρ* then statistically they are independent, that is, one could consider them separately. The 3 distinct areas of high abundance remaining in the lower panel of Fig 7 are all separated by at least 2*ρ*, quite a large value given what we know of cod mobility [59–61]. One might expect an element of isolation by distance [62] and therefore divergence—hence an argument for separate sub-populations. The physical separation argues for the treatment of these 3 remnants as distinct putative sub-populations. From a conservancy perspective we would argue for the desirability of treatment of the aggregations as biologically independent unless other information comes to light. It is important to remember that the model does not capture within year movement patterns (that is, is based only on the July survey data: the fish from these spatial aggregations present in July could mix at a different time of year) so this is not definitive, only suggestive, but does concur with previous categorizations and it is known that cod display high degrees of site fidelity [59–61, 63]. As an item for further work one could postulate that overfishing has resulted in the removal of cod from the Banquereau Bank (between the eastern 2 areas in Fig 7) resulting in the division of the previous population into 2 distinct remnants [20]. Previous studies [64] found that median distance travelled to recapture for cod in this area of the Scotian shelf was 36 kms; our work supports the contention that the remnant patches are even less connected that they once were due to the erosion and elimination of some subpopulations, notably Banquereau bank. Recolonization of such a vacant, yet previously dense, portion of the range would be a hallmark of any recovery. Indeed, the spatial distribution of the cod throughout the 1990’s shows little variation and the stock remains at low abundance, that is ‘fails to recover’.

**Fig 8:**
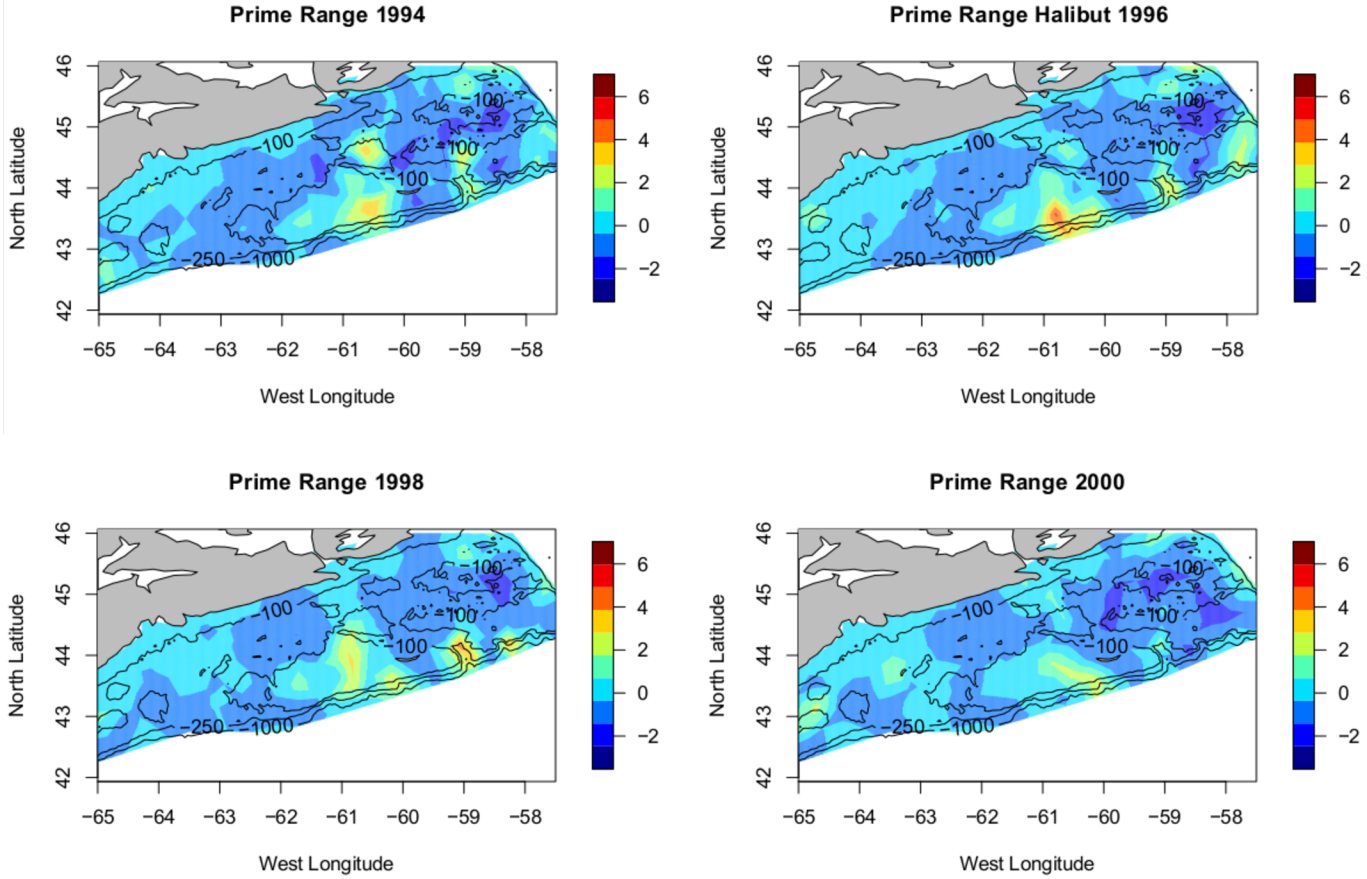
Failure to recover. Plot of the posterior mean for the years 1994 through 2000, showing only those areas where the predicted mean is > 75th percentile. Viewed as ‘core range’. The species range fails to recover abundance and the habitat remains unchanging.

The premise here is that the range of a species is density dependent—that is, they only spread out when their prime territory reaches maximum capacity, or, conversely, that the density of the species will remain relatively constant in the most suitable habitat and that increases in total abundance will increase the total range and *not* the density [18]. If the total abundance of the species is reduced the total range may contract but density sampled in the prime territory may not change at all since there is an in-migration effect. The reaction in abundance through the years of collapse should be seen in our models posterior mean not as a reduction of the maximum level but as a shrinking of the area of maximal (or simply high) abundance. That is, we should see range shrink, not maxima. Examining Figs 7 and 8, this is exactly what we do see. We do not conclude directly that these areas of remaining high relative abundance are therefore prime habitat for cod. Since we believe the cod have been removed through overfishing [13, 15, 19] and since we do not know the rate at which the cod will in-migrate to fill their now vacant former habitat [20, 65] we conservatively interpret *ρ* as the range of spatial aggregation of the remnants of the original population. These remnants are, now, the sole potential source for recolonization of any formerly important habitat vacated by overfishing and, this recolonization will be seen as a reversal of the trends noted herein; an increase in the area of moderate density for cod. This suggests that one indicator of recovery for the Scotian shelf Atlantic cod would be an increase in their abundance *outside* of the areas noted as containing the remnant sub-populations and argues strongly for the managerial efforts to sustain cod recovery protect these areas important to the remnant sub-populations. Indeed, the recolonization of these areas by cod and the recovery of the stock are synonymous. The failure to recover seen in Atlantic cod [17] is evident in Fig 7, the cod do not expand from their remnant sub-populations. In any event, the survival of the Atlantic cod depends on the future of these three surviving remnant sub-populations and knowing their location and extent is valuable information to any management plan.

**Fig 9:**
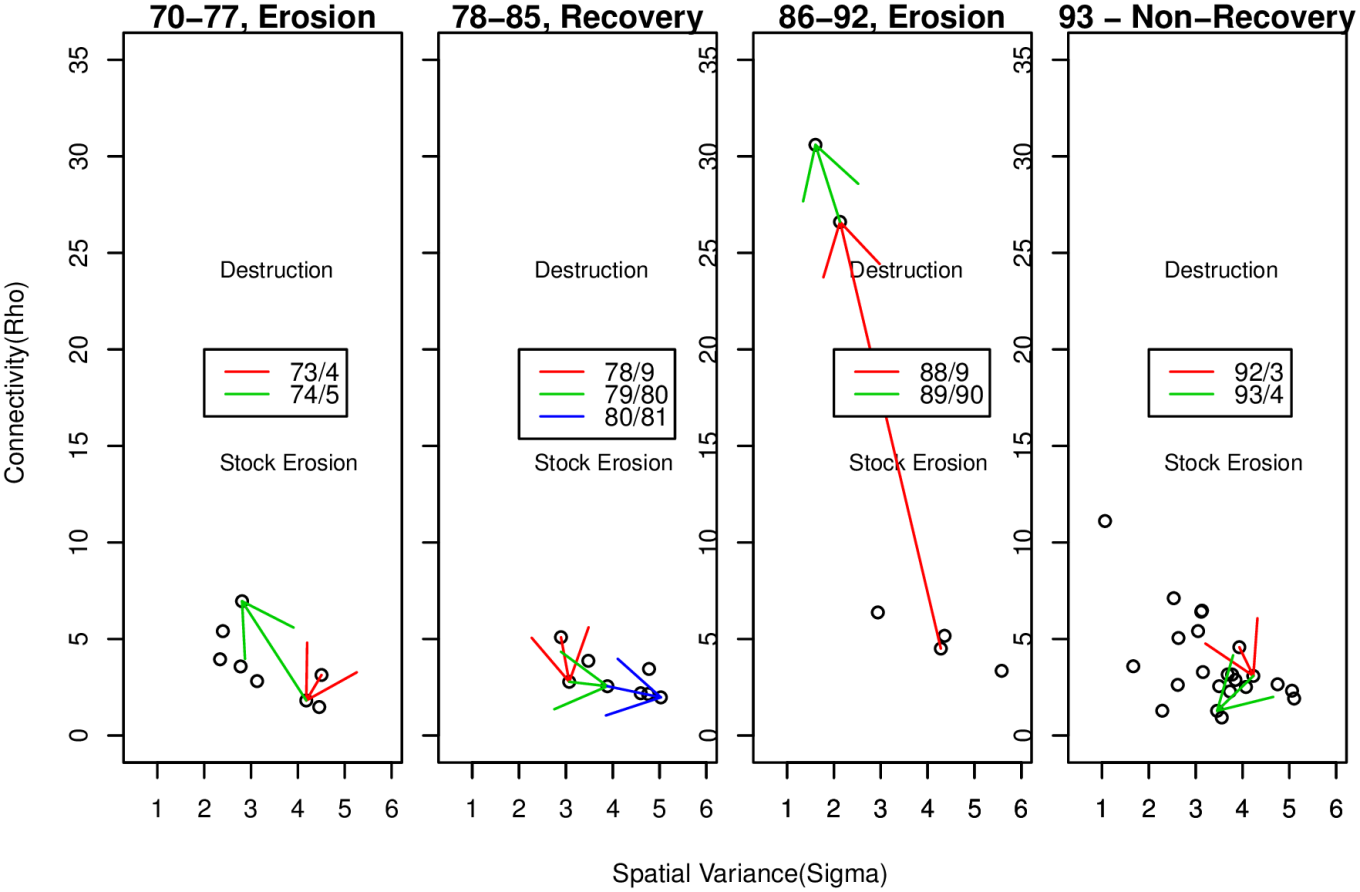
Observed parameter movements. What the parameters were observed to do. The coloured arrows correspond to the years discussed in Figs 10 and 11.

Ideally, a well managed stock should not suffer changes in distribution or structure due to exploitation. In the case of Atlantic cod this was definitely not the case; measuring CPUE only in places of relatively high abundance failed to detect the contraction of a depleting stock onto core range until it was too late to prevent the collapse of the stock, resulting in the near disappearance of cod in parts of the Scotian Shelf such as Banquereau bank. Both the distribution, and structure were changed. This leads us to wonder how we may detect such changes in structure using our techniques. To do so we fit our model to the data on a year to year basis, and our findings are displayed in Fig 9, in which we display the joint behaviour of *ρ* and *σ* in four panels; one for each of the periods identified above, with some years of notable change highlighted. Small values of *ρ* are at the top, indicating high structure, small values of *ρ* are at the bottom, indicating lack of structure (flatness). Examining this figure we note the following:

1. In the first panel, 1970-1977, in the period which we term the ‘first collapse’ we see the very large shift of the parameters to the lower left marking the partial collapse of 1975.
2. The second panel, 1978-1985, might be termed the ‘first recovery’. Canada imposes a 200 mile EEZ and the cod see some relief from fishing pressure. We see the cod regain first structure, 1978-1979, then start to regain abundance, 1979-1980 and 1980-1981. the re-establishment of structure is what we might expect under conditions of DDHS, a return to prime range. The recovery of numbers seems to lag re-establishment of structure.
3. The third panel, the ‘second collapse’. From 1988 to 1989, and again from 1989 to 1990, there is an even stronger movement to the lower left, i.e. a simultaneous increase in *ρ* and decrease in *σ*. While the moratorium was imposed in the early 1990s few would that it was imposed too late to avert significant degradation of the stock and Atlantic cod suffered a more profound collapse than that of 1975. Our analysis shows that the real damage was inflicted 1988-1991.
4. Panel four. After the imposition of the moratorium in the early 1990s we see, not recovery, but a period of what might be termed stable non-recovery. We see perhaps an effect of the imposition of the moratorium on Atlantic Cod, but, a re-establishment of structure without an increase in numbers. An expression of DDHS, the remnant fish re-aligning themselves onto the available habitat. This doesn’t really constitute a ‘recovery’ however. It only reaches the top center of the plot and *σ* remains small. Contrast this to the recovery of the early 80s where there is bias to the right of the plot. This top center position is the new reality for cod, stable non-recovery.

**Fig 10:**
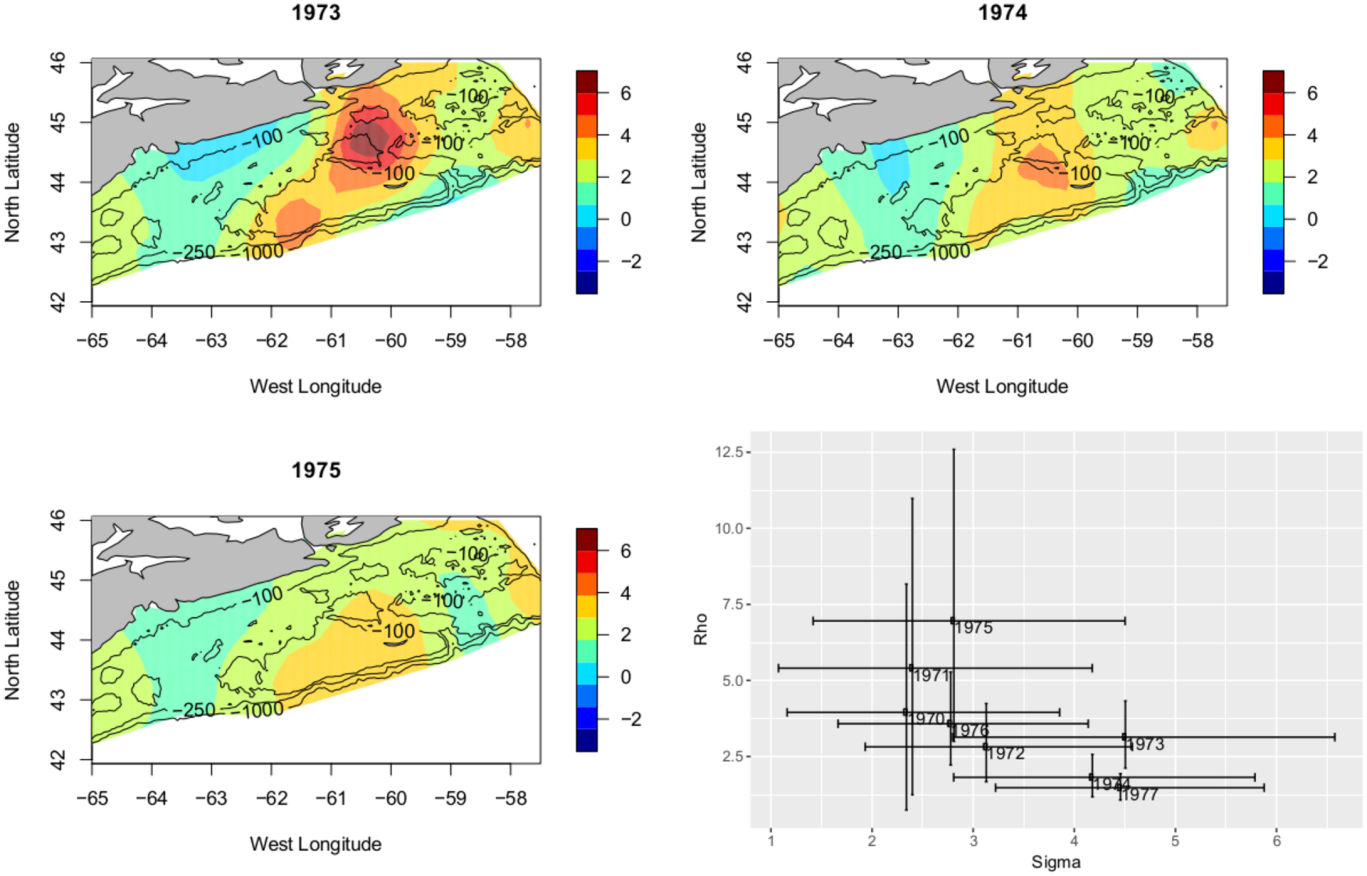
The ‘first collapse’. Plot of the posterior mean for the years 1973 through 1975, showing a dramatic flattening of the RF. This is indicative of circumstance in which heaving fishing was *eroding* the structure of the cod distribution leading to a partial collapse. In this period, *ρ* = 3.1 → *ρ* = 2.61 → *ρ* = 7.50 *σ* = 3.7 → *σ* = 2.8 → *σ* = 2.2. A simultaneous large increase in *ρ* and decrease in *σ*.

**Fig 11:**
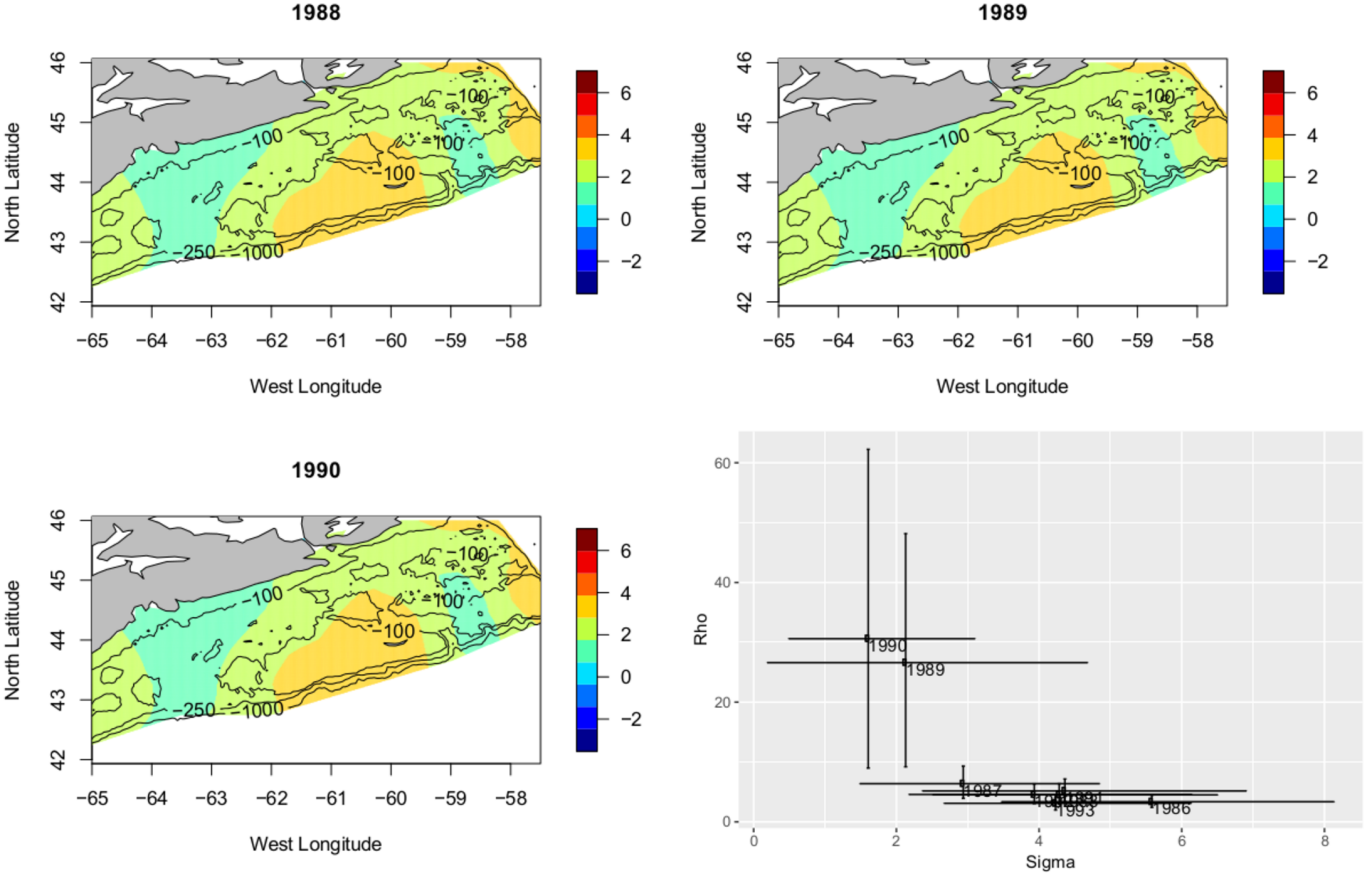
The ‘second collapse’. Plot of the posterior mean for the years 1988 through 1990, showing a dramatic flattening of the RF. This is indicative of circumstance in which heavy fishing was *effectively destroying* the structure of the cod distribution leading to near total collapse. In this period the flattening is more severe than what was seen in the 1970s, *ρ* = 4.40 → *ρ* = 26.43 → *ρ* = 28.6 *σ* = 4.2 → *σ* = 02.1 → *σ* = 1.6. A simultaneous *very* large increase in *ρ* and decrease in *σ*. the collapse of the early 1990s is much more pronounced.

We look first at the ‘first collapse’, particularly the period 1973-1975. Focussing on the first collapse, the predicted spatial mean from 1973-1975 shows erasure of areas of high density and a corresponding flattened RF, increase in *ρ* and decrease in *σ*. This is a clear picture of spatial erosion. While there is uncertainty in the parameter values during the ‘first collapse’, the pattern of parameter behaviour is repeated even more strongly in the ‘second collapse’. Fig 11 displays the predicted spatial mean over the period 1988-1990. Instead of the partial collapse seen in the 1970s the Atlantic cod hits historic lows across the Scotian Shelf. The erosion, perhaps destruction is not too strong a word, of the cod is seen as an even more extreme flattening of the RF, with correspondingly larger increase in *ρ* and decrease in *σ*. Examined in detail these collapses display common trends in parameters revealing erosion of the spatial structure present in the Atlantic cod.

**Fig 12:**
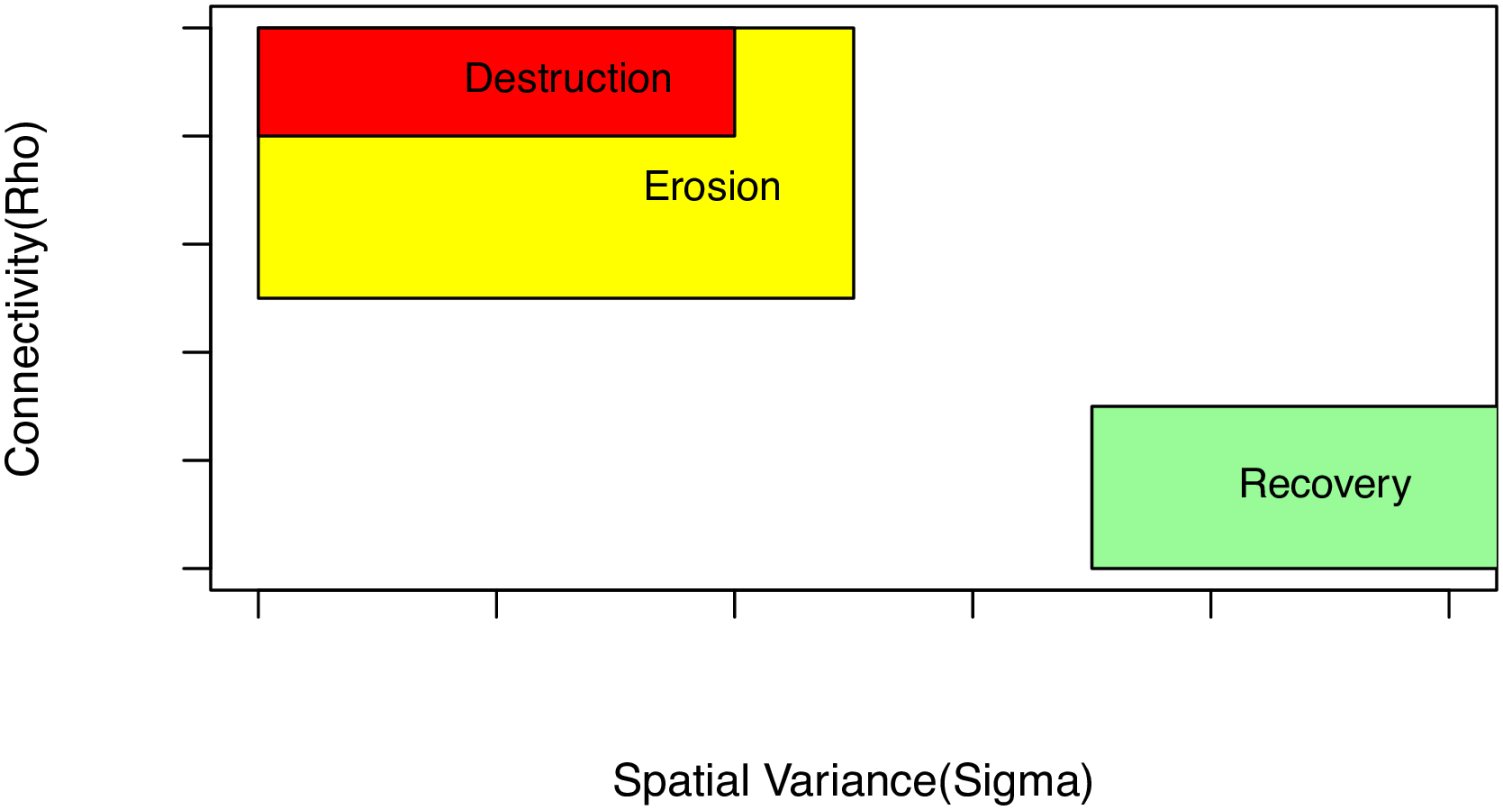
Schematic of parameter behaviour. A schematic picture of expected parameter values under different conditions in the fishery. Note that large *ρ* values are at the bottom of the schematic.

Under the conditions prevailing in the different periods discussed here, what should we have *expected* our parameters to do? Suppose there were no fish, that is, abundance was 0 everywhere. What would our parameters show? Our spatial connectivity parameter, *ρ*, would be +∞ since the field is 0 everywhere, no matter how far separated. Now, practically, our estimate will be some finite number since we are estimating in a finite space, but $\hat{\rho }$ will be large. On the other hand, *σ*, the variance, would be 0 since the field is everywhere 0. Taken *in isolation*, *σ* is fairly easy to interpret. Since in our modelling framework our model of the spatial covariance structure is the RF; if there is wide separation between areas of high predicted Cod abundance and low predicted abundance, *σ* will be large. So, *in isolation*, a large *σ* is needed when there is lots of contrast between areas of high fish density and low density, a small *σ* will mean that density is constant or nearly so over the space. In Fig 12 we provide a schematic view of expected parameter behaviour under differing conditions:

- We expect to see some fluctuation in the parameter values. Horizontal noise in *σ* is normal, year to year, fluctuation in fish numbers. Vertical noise in *ρ* is analogous variation in our estimation of structure.
- We expect an erosion of, or loss of, structure in the stock to express as an increase in the value of *ρ*, a flattened structure results in an increase in the spatial measure of covariance.
- A species experiencing moderate, or well managed, fishing might be expected to see a small reduction in *σ* compared to the unexploited state, with little change in *ρ*.
- A species experiencing ‘recovery’, will see a simultaneous re-establishment of structure and increase in numbers; this would imply a movement to the top right of Fig 12.
- Large movement to lower left correlates to stock **destruction** as the densest areas of fish are removed and the stock structure is removed. Essentially, the field we are modelling is being flattened under stress due to over-fishing (i.e. the extreme depletion, or utter removal, of fish in high density areas), resulting in the *simultaneous* reduction of *σ* and increase in *ρ*, that is a large movement to the lower left on Fig 12.

### Future directions

This analysis opens up a number of possibilities, and questions. From the practical point of view of stock manager models like this suggest that, in addition to traditional means of monitoring the health of a subject stock one might routinely examine these parameters looking for large movements or trends. Notably, for instance, an exploited stock should seek to avoid movement to the lower left of the plot, when depletion begins to erode the stock structure there is damage being done. Conversely, stock which one hopes to see recover might be monitored for movement to the upper right as being encouraging. Now these parameters are always going to be subject to some fluctuation from normal fluctuation in the spatial distribution and abundance of the subject stock, but, one can imagine that there exists a ‘natural value’ for them, i.e. that they will have some sort of true mean upon which they will centre in a population free from external disturbances. If you will, a box at the top center of the *ρ*, *σ* plot will bound ‘good’ combinations of parameters. For the manager, excursions from the box require explanations.

## References

[1] Gaston KJ, Blackburn TM. Pattern and process in macroecology. Blackwell Science; 2000.

[2] Gaston KJ, Blackburn TM, Greenwood JJD, Gregory RD, Quinn RM, Lawton JH. Abundance-Occupancy Relationships. Journal of Applied Ecology. 2000;Vol. 37 S1, 39–59. 10.1046/j.1365-2664.2000.00485.x

[3] Fretwell SD, Lucas HL. On territorial behaviour and other factors influenceing the habitat distribution of birds. I. Theoretical developement. Acta Biotheoretica. 1970;Vol. 19, 16–36. 10.1007/BF01601953

[4] Moilanen A, Hanski I. Metapolulation dynamics: effects of habitat quality and landscape structure. Ecology. 1998;Vol. 79(7), 2503–2515. 10.1890/0012-9658(1998)079[2503:MDEOHQ]2.0.CO;2

[5] Maury O, Gascuel D. ‘Local overfishing’ and fishing tactics: theoretical considerations and applied consequences in stock assessment studied with a numerical simulator of fisheries. Aquat Living Resour. 2001;Vol. 14, 203–210. 10.1016/S0990-7440(01)01115-9

[6] Frank KT, Brickman DW. Allee effects and compensatory population dynamics within a stock complex. Can J Fish Aquat Sci. 2000;Vol. 57(3), 513–517. 10.1139/f00-024

[7] Hauser L, Carvalho G. Paradigm shifts in marine fisheries genetics: ugly hypotheses slain by beautiful facts. Fish and Fisheries. 2008;Vol. 9(4), 333–362. 10.1111/j.1467-2979.2008.00299.x

[8] Kerr L, Cadrin S, Secor D. The role of spatial dynamics in the stability, resilience and productivity of an estuarine fish population. Ecological Application. 2010;Vol. 20(2), 497–507. 10.1890/08-1382.1 20405802

[9] Cianelli L, Fisher J, Skern-Mauritzen M, Hunsicker M, Hidalgo M, Frank K, et al Theory, consequences and evidence of eroding population spatial structure in harvested marine fishes: a review. Marine Ecology Progress Series. 2013;Vol. 480, 227–243. 10.3354/meps10067

[10] Corten A. Recruitment depressions in North Sea herring. ICES J Mar Sci. 2013;Vol. 70, 1–15. 10.1093/icesjms/fss187

[11] Ruzzante D, Mariani S, Bekkevold D, Andre C, Mosegaard H, Clausen L, et al Biocomplexity in a highly migratory pelagic marine fish, Atlantic herring. Proceedings of the Royal Society. 2006;Series B-Biological Sciences 273: 2279–2284. 10.1098/rspb.2005.3463 PMC1560315 16777738

[12] Safina C, Rosenberg A, Myers R, Q T II, Collie J. U.S. Ocean Fish Recovery: Staying the course. Science. 2005;Vol. 309, 707–708. 10.1126/science.1113725 16051773

[13] Hutchings J, Myers R. What can be learned from the collapse of a renewable resource? Atlantic cod, Gadus morhua, off Newfoundland and Labrador. Can J Fish Aquat Sci. 1994;Vol. 51, 2126–2146. 10.1139/f94-214

[14] Walters C, Maguire J. Lessons for stock assessment from the northern cod collapse. J Rev Fish Biol. 1996;Vol. 6, 125–137.

[15] Myers R, Hutchings J, Barrowman N. Why do fish stocks collapse? The example of cod in Atlantic Canada. Ecological Applications. 1997;Vol. 7(1), 91–106. 10.1890/1051-0761(1997)007[0091:WDFSCT]2.0.CO;2

[16] Rose G, deYoung B, Kulka D, Goddard S, Fletcher G. Distribution shifts and overfishing the northern cod (Gadus morhua): a view from the ocean. Can J Fish Aquat Sci. 2000;Vol. 57, 644–663. 10.1139/f00-004

[17] Fu C, Mohn R, Fanning LP. Why the Atlantic cod (Gadus morhua) stock off eastern Nova Scotia has not recovered. Can J Fish Aquat Sci. 2001;Vol. 58, 1613–1623. 10.1139/f01-095

[18] Rose GA, Dutka DW. Hyperaggregation of fish and fisheries: how catch-per-unit-effort increased as the northern cod (Gadus morhua) declined. Can J Fish Aquat Sci. 1999;Vol. 56(Suppl. 1), 118–127. 10.1139/f99-207

[19] Hutchings J. Spatial and temporal variation in the density of northern cod and a review of hypotheses for the stock’s collapse. Can J Fish Aquat Sci. 1996;Vol. 53, 943–962. 10.1139/f96-097

[20] Shackell NL, Frank KT, Brickman DW. Range contraction may not always predict core areas: an example from marine fish. Ecological Applications. 2005;Vol. 15(4), 1440–1449. 10.1890/04-0642

[21] Swain D, Wade E. Density-dependent geographic distribution of Atlantic cod (Gadus Morhua) in the southern Gulf of St. Lawrence. Can J Fish Aquat Sci. 1993;Vol. 50(4) 725–733. 10.1139/f93-083

[22] Tamdrari H, Castonguay M, Brethes J, Duplisea D. Density-independent and -dependent habitat selection of Atlantic cod (Gadus Morhua) based on goeostatistical aggregation curves in the northern Gulf of St. Lawrence. ICES J Mar Sci. 2010;Vol. 67, 1676–1686. 10.1093/icesjms/fsq108

[23] Swain DP, Wade EJ. Density dependent Geographic distribution of atlantic cod (Gadus Morhua) in the Southern Gulf of St. Lawrence. Can J Fish Aquat Sci. 1993;Vol. 50, 725–733. 10.1139/f93-083

[24] Fisher JAD, Frank KT. Abundance-distribution relationships and conservation of exploited marine fishes. Marine Ecology Progress Series. 2004;Vol. 279, 201–213. 10.3354/meps279201

[25] Rose GA, deYoung B, Kulka D, Goddard S, Fletcher G. Distribution shifts and overfishing the northern cod (Gadus Morhua): a view from the ocean. Can J Fish Aquat Sci. 2000;Vol. 57(3), 644–663. 10.1139/f00-004

[26] Reuchlin-Hugenholtz E, Shackell NL, Hutchings JA. The Potential for Spatial Distribution Indices to Signal Thresholds in Marine Fish Biomass. PLOS ONE. 2015; 10.1371/journal.pone.0120500 25789624 PMC4366403

[27] Horsman TL, Shackell NL. Atlas of important habitat for key fish species of the Scotian Shelf, Canada. DFO Can Tech Rep Fish Aquat Sci. 2009;2835.

[28] Ricard D, Shackell NL. Population status (abundance/biomass, geographic extent, body size and condition), important habitat, depth, temperature and salinity preferences of marine fish and invertebrates on the Scotian Shelf and Bay of Fundy (1970-2012). DFO Can Tech Rep Fish Aquat Sci. 2013;3012.

[29] Smith CD, King MC, Shackell NL. Spring, summer and fall seasonal survey maps of fish distribution on the Scotian Shelf between 1978 and 1984. Cdn Manuscript Rpt of Fish and Aquat Sci. 2013;3013.

[30] OBIS. Ocean Biogeographic Information System: DFO Scotian Summer Research Trawl Survey (OBIS Canada); 2014. Available from: http://www.gbif.org/dataset/8393f330-f762-11e1-a439-00145eb45e9a.

[31] Waters C, Maguire J. Lessons for stock assessment from the northern cod collapse. Reviews in Fish Biology and Fisheries. 1996;Vol. 6, 125–137.

[32] Amante C, Eakins BW. ETOPO1 1 Arc-Minute Global Relief Model: Procedures, Data Sources and Analysis. NOAA Technical Memorandum NESDIS NGDC-24. 2009;1–19.

[33] Cressie N, Wilkie CK. Statistics for spatio-temporal data. Wiley; 2011.

[34] Thorson JT, Shelton AO, Ward EJ, Skaug HJ. Geostatistical delta-generalized linear mixed models improve precision for estimated abundance indices for West Coast groundfishes. ICES Journal of Marine Science. 2014;.

[35] Shelton AO, Thorson JT, Ward EJ, Feist BE. Spatial semiparametric models improve estimates of species abundance and distribution. Can J Fish Aquat Sci. 2014;Vol. 71 1655–1666. 10.1139/cjfas-2013-0508

[36] Rue H, Martino S, Chopin N. Approximate Bayesian inference for latent Gaussian models using integrated nested Laplace approximations. Journal of the Royal Statistical Society. 2009;Series B, Vol. 71, No. 2, 319–392. 10.1111/j.1467-9868.2008.00700.x

[37] Lindgren F, Rue H. Bayesian Spatial Modelling with R-INLA. Journal of Statistical Software. 2015;Vol. 63, No. 19 10.18637/jss.v063.i19

[38] Lindgren F, Rue H, Lindstrom J. An explicit link between Gaussian fields and Gaussian Markov random fields: The SPDE approach (with discussion). Journal of the Royal Statistical Society. 2011;Series B, Vol. 73, No. 4, 423–498. 10.1111/j.1467-9868.2011.00777.x

[39] Illian JB, Sørbye SH, Rue H, Hendrichsen D. Using INLA to fit a Complex Point Process Model with Temporally Varying Effects—A Case Study. Journal of Environmental Statistics. 2012;Vol 3, No. 7.

[40] R Core Team. R: A Language and Environment for Statistical Computing. Vienna, Austria; 2013 Available from: http://www.R-project.org.

[41] Carson S, Flemming JM. Seal Encounters at Sea: A Contemporary Spatial Approach using R-INLA. Ecological Modelling,. 2014;Vol. 291, 175–181. 10.1016/j.ecolmodel.2014.07.022

[42] Munoz F, Pennino MG, Conesa D, López-Quílez A, Bellido JM. Estimation and prediction of the spatial occurrence of fish species using Bayesian latent Gaussian models. Stochastic Environmental Research and Risk Assessment. 2013;Vol. 27, No. 5, 1171–1180. 10.1007/s00477-012-0652-3

[43] Quiroz ZC, Prates MO, Rue H. A Bayesian Approach to Estimate the Biomass of Anchovies Off the Coast of Perú. Biometrics. 2014;. 25257036 10.1111/biom.12227

[44] Pennino MG, Munoz F, Conesa D, López-Quílez A, Bellido JM. Modelling Sensitive Elasmobranch Habitats. Journal of Sea Research. 2013;Vol. 83, 209–218. 10.1016/j.seares.2013.03.005

[45] Paradinas I, Conesa D, Pennino MG, Munoz F, fernandez AM, Lopez-Quilez A, et al Bayesian spatio-temporal approach to identifying fish nurseries by validating persistence areas. Marine Ecology Progress Series. 2015;Vol. 528, 245–255. 10.3354/meps11281

[46] Pennino MG, Munoz F, Conesa D, López-Quílez A, Bellido JM. Bayesian Spatio-Temporal Discard Model in a Demersal Trawl Fishery. Journal of Sea Research. 2014;Vol. 90, 44–53. 10.1016/j.seares.2014.03.001

[47] Cosandey-Godin A, Krainski ET, Worm B, Flemming JM. Applying Bayesian spatio-temporal models to fisheries bycatch in the Canadian Arctic. Can J Fish Aquat Sci. 2015;Vol. 72(2) 186–197. 10.1139/cjfas-2014-0159

[48] Zuur A, Ieno EN, Walker N, Saviliev AA, Smith GM. Mixed Effects Models and Extensions in Ecology with R. Springer; 2009.

[49] Hastie TJ, Tibshirani RJ. Generalized Additive Models. Chapman & Hall; 1990.

[50] Taylor BM, Diggle PJ. INLA or MCMC? A Tutorial and Comparitive Evaluation for Spatial Prediction in log-Gaussian Cox Processes. http://arxivorg/abs/12021738v2. 2012;.

[51] Simpson D, Lindgren F, Rue H. In order to make spatial statistics computationally feasible, we need to forget about the covariance function. Environmetrics. 2012;Vol. 23, 65–74. 10.1002/env.1137

[52] Simpson D, Lindgren F, Rue H. Think continuous: Markovian Gaussian models in spatial statistics. Spatial Statistics. 2012;Vol. 1, 16–29. 10.1016/j.spasta.2012.02.003

[53] Cameletti M, Ignaccolo R, Bande S. Comparing spatio-temporal models for particulate matter in the piemonte. Environmetrics. 2011;Vol. 22, 985–996. 10.1002/env.1139

[54] Cameletti M, Lindgren F, Simpson D, Rue H. Spatio-temporal modeling of particulate matter concentration through the SPDE approach. Advances in Statistical Analysis. 2013;Vol. 97, No. 2, 109–131. 10.1007/s10182-012-0196-3

[55] Simpson DP, Rue H, Martins TG, Riebler A, Sorbye SH. Penalising model component complexity: A principled, practical approach to constructing priors. 2015;Available from: http://https://arxiv.org/abs/1403.4630.

[56] Spiegelhalter DJ, Best NG, Carlin BR, van der Linde A. Bayesian measures of model complexity and fit. Journal of the Royal Statistical Society. 2002;Series B, Vol. 64, No. 4, 583–639. 10.1111/1467-9868.00353

[57] Geisser S, Eddy WF. A Predictive Approach to Model Selection. Journal of the American Statistical Association. 1979;Vol. 74, 153–160 (Corr: V75, p765). 10.1080/01621459.1979.10481632

[58] Campana SE, Mohn RK, Smith SJ, Chouinard GA. Spatial implications of a temperature-based growth model for Atlantic cod (Gadus morhua) off the eastern coast of Canada. Can J Fish Aquat Sci. 1995;Vol. 52, 2445–2456. 10.1139/f95-835

[59] Hedger R, McKenzie E, Heath M, Wright P, Scott B, Gallego A, et al Analysis of the spatial distributions of mature cod (Gadus morhua) and haddock (Melanogrammus aeglefinus) abundance in the North Sea (1980-1999) using generalised additive models. Fisheries Research. 2004;Vol. 70, 17–25. 10.1016/j.fishres.2004.07.002

[60] Svedäng H, Bardon G. Spatial and temporal aspects of the decline in cod (Gadus morhua L.) abundance in the Kattegat and eastern Skagerrak. ICES J MAR SCI. 2003;Vol. 60, 32–37. 10.1006/jmsc.2002.1330

[61] Wright PJ, Galley E, Gibb IM, Neat FC. Fidelity of adult cod to spawning grounds in Scottish waters. Fisheries Research. 2006;Vol. 77, 148–158. 10.1016/j.fishres.2005.10.008

[62] Cadrin SX, Kerr LA, Mariani S. Stock Identification Methods (2nd ed.). Academic Press; 2014.

[63] Reubens JT, Pasotti F, Degraer S, Vincx M. Residency, site fidelity and habitat use of Atlantic cod (Gadus morhua) at an offshore wind farm using acoustic telemetry. Marine Environmental Research. 2013;Vol. 90, 128–135. 10.1016/j.marenvres.2013.07.001 23937893

[64] Shackell NL, Stobo WT, Frank KT, Brickman DW. Growth of cod (Gadus morhua) estimated from mark recapture programs on the Scotian shelf and adjacent areas. ICES Journal of Marine Science. 1997;Vol. 54, 383–398. 10.1006/jmsc.1996.0173

[65] Erisman BE, Allen LG, Claisse JT, II DJP, Miller EF, Murray JH. The illusion of plenty: hyperstability masks collapses in two recreational fisheries that target fish spawning aggregations. Can J Fish Aquat Sci. 2011;Vol. 68, 1705–1716. 10.1139/f2011-090

